# A New Method to Interpret Cluster Analysis Results in the Presence of Heterogeneous Clusters

**DOI:** 10.17505/jpor.2026.29047

**Published:** 2026-03-26

**Authors:** András Vargha, Regina Postáné Török

**Affiliations:** 1 Institute of Psychology, Károli Gáspár Reformed Church University, Budapest, Hungary; 2 Institute of Psychology, Eötvös Loránd University, Budapest, Hungary

**Keywords:** typical cluster members, atypical cluster members, cluster analysis, ROPstat, parental attachment types

## Abstract

Contrary to several other statistical analyses (ANOVA, linear regression, etc.) normality is not a requirement of cluster analysis (CA). However, certain types of departure from normality, such as high skewness, can cause problems in CAs. In such cases the proportion of extreme cases will increase, increasing the chance to obtain heterogeneous clusters. The aim of the paper is to propose a new method for interpreting CA results in the presence of heterogeneous clusters. After performing CA, all cases are classified as either typical or atypical, depending on how close they are to their own cluster center (cluster centroid). A key concept is a new variable that measures the distance of each case to its own cluster centroid. A case is considered typical if this distance does not exceed a predetermined threshold, and atypical if it does. Typical cases can be used to provide a robust estimation of the cluster centroids. Additionally, analyzing subgroups of atypical cases within clusters where they reach an interpretable proportion can refine the explanation of cluster profile. The usefulness of the new method is demonstrated using four parental attachment variables of avoidance and anxiety, where high skewness and therefore heterogeneous clusters are anticipated. The study sample consisted of 918 young adults aged between 20 and 35. Standard hierarchical and *k*-means clustering analysis identified a 6-cluster structure as the best solution, yielding easily interpretable parental attachment types. The proportion of atypical cases exceeded 5% in three clusters. The psychological meaning of these clusters could be explored in more detail by computing cluster centroids based on typical cases and then comparing groups of typical and atypical cases using Welch’s *t*-tests. The new method can easily be applied in the *Validation* module of the latest version of the ROPstat software. The parental attachment styles explored were comparable to those found in literature.

## Introduction

Normality is an important assumption of several statistical analyses such as *t*-tests and ANOVAs, regression analysis, factor analysis, etc. (Scheffé, 1959; Winer, 1971; Tabachnick & Fidell, 2013). Cluster analysis (CA) is an exception because if the multivariate distribution of variables in CA is normal, there will only be one center (Tong, 2012) and therefore different clusters with well-identified centroids cannot be formed.

However, certain types of departure from normality, such as high skewness, can cause problems in CAs (Christiansen, 2007; Rousseeuw & Kaufman, 2009; Wallace et al., 2018). To tackle the problem of heavy skewness, it is suggested that either the variables submitted to CA are transformed (Meredith et al., 2007; Bennett et al., 2008; Melnykov & Zhu, 2019) or some kind of robust CA is used (Rousseeuw & Kaufman, 2009; Gallaugher et al., 2020; Liu et al., 2021; Schroth & Muma, 2021; Gallaugher et al., 2022; Dang et al., 2023).

Attachment variables in psychology are often skewed (Raja et al., 1992; Smallbone & Dadds, 2000; Meredith et al., 2006; Bennett et al., 2008; Condon et al., 2008). For example, Vargha and Grezsa (2025) used data on parental attachment in a male sample of Hungarian adolescents to explore the main attachment types using CA. The variables were the four attachment scales of the mother and father domains (Mother avoidance, Mother anxiety, Father avoidance, Father anxiety) of the Experiences in Close Relationships – Relationship Structures Questionnaire (ECR-RS; Fraley et al., 2011). The anxiety scales were so strongly skewed that their medians equaled the minimum score (Vargha & Grezsa, 2024).

In a recent article Moeller (2025) draws attention to several pitfalls of *z*-standardization in CA even when the variables have different scales. In such cases some type of transformation (so called normalization) is necessary, to create a common scale of the variables, but according to Moeller (2025) the *z*-standardization is a bad choice. Dudek and Walesiak (2020) suggested 18 methods of variable normalization for this purpose, where *z*-standardization belongs to the type of normalization with a measure of sample variability (standard deviation or median absolute deviation). If normalization is necessary, Moeller (2025) suggests a method based on the range, the Proportion of Maximum Scaling Transformation (POMS). However, if the variables are normalized (e.g., z-standardized) separately and differently before CA, the transformed variables will lose their original scale units. This has a significant impact on both the CA results and the interpretability of the clusters explored. According to Moeller (2025), it is advisable not to standardize the variables before conducting a CA. And, as Vargha (2025) demonstrates, simple linear transformations are often sufficient to bring the input variables to a common scale even if their original scales are different.

If a variable is heavily skewed, it will have a long tail, with increased distances between cases in this part of the distribution. The increased distances may yield heterogeneous clusters, which impairs the overall homogeneity of the cluster structure.

The aim of the present paper is to propose a method for improving the interpretation of cluster analysis results in the presence of heterogeneous clusters. The key concept is a new variable that measures how far each case is from its own cluster center. We note that in the *k*-means analysis of SPSS (IBM Corp., 2020) this variable can be computed and saved for each case. A case is considered *typical* if this distance does not exceed a predetermined threshold, and *atypical* if it does. If the input variables are standardized, the suggested threshold value is 0.70, which is the lower threshold for being considered extreme in residual analysis (Bergman, 1988; Bergman et al., 2003; Vargha et al., 2015). If the variables are not standardized, the suggested threshold is 0.70 times *Var_t_*, the mean variance of the variables submitted to CA. It is important to note that the suggested threshold of 0.70 is not compulsory. Increasing this threshold will decrease the proportion of atypical cases, and vice versa. Based on this categorization of the classified cases (typical vs atypical) the interpretation of the obtained cluster structure can be improved in two ways.

1. Compute and evaluate cluster centroids restricted to only typical cases. This is analogous to compute trimmed means instead of means, which yields robust sample centers (Wilcox, 1996, 1997). Centroids based on typical cases may better represent the explored types represented by the clusters. And the user can specify a threshold for the percentage of atypical cases, like the trimming percentage in computing trimmed means.
2. Meaningful subgroups may be identified by comparing typical and atypical cases within clusters where atypical cases occur in substantial number, which also refines the interpretation of the cluster results.

The usefulness of this new method will be demonstrated using parental attachment variables, where high skewness and therefore heterogeneous clusters are anticipated.

## A Study Exploring Parental Attachment Types

### Method of ECR-RS scale reduction

In our study, we planned to use a shortened version of the Hungarian ECR-RS attachment questionnaire (Jantek & Vargha, 2016). In ECR-RS attachment is measured using two dimensions (avoidance and anxiety), with a secure attachment falling at the lower end of each dimension (Fraley et al., 2011). Avoidance scales are formed from six items, anxiety scales from three items for four domains (mother, father, intimate partner, and friend). We only reduced the number of items of the avoidance scales, from six to three. Among the six avoidance items the first four are reversed and the last two are direct (Fraley et al., 2011). We retained the two direct items and, from the reversed items, selected the one showing the highest correlation with the entire avoidance scale. Computations were performed on a separate sample of size *N* = 336 from the study of Jantek and Vargha (2016) for the four domains separately. In all cases, item 2 ('I usually discuss my problems and concerns with this person') produced the largest correlation. Notably, it also had the largest item-remainder correlation in the item analysis of the avoidance scale across all four domains. Therefore, the reduced avoidance scale comprised items 2, 5 and 6, and in our recent empirical study the ECR-RS was administered with three items for each of the avoidance and anxiety scales in all domains.

### Participants and procedure

Participants recruited from Hungary completed a 155item online questionnaire. Ethical approval for the study was granted by the Research Ethics Committee of Károli Gáspár University of the Reformed Church (permission number: BTK/2318-1/2023). Participation was voluntary and anonymous. Informed consent was obtained but no compensation was given. The study sample consisted of 918 persons (388 males and 528 females, two persons did not report their gender; age mean: 30.48 years, *SD* = 3.83, Min = 20, Max = 35).

### Variables

The overarching aim of the study was to investigate the relationship between attachment and the turnover intention among young white-collar workers. Within this framework we focused on parental attachment variables of Mother avoidance (AvoidMo), Mother anxiety (AnxMo), Father avoidance (AvoidFa), and Father anxiety (AnxFa), as defined above in the shortened ECR-RS. For each item respondents had to rate on a seven-point Likert scale how much, in their view, they agreed with the statement corresponding to that item (1 = Strongly disagree; 7 = Strongly agree). Each scale consisted of three items and only the first item on both avoidance scales was a reverse item. The four scales were created by computing an average of the items that corresponded to them.

### Statistical analysis

Firstly, we checked the factor structure of the four parental scales of the newly shortened ECR-RS via confirmatory factor analysis (CFA) and computed Cronbach’s alpha and McDonald's omega reliability measures to assess the internal consistency of the four scales, since the reliability of input variables is an important prerequisite for exploring good cluster structures (Vargha & Bergman, 2019; Gergely & Vargha, 2021).

Secondly, we conducted standard hierarchical and *k*means CAs to explore a statistically adequate and theoretically acceptable cluster structure of parental attachment.

Thirdly, we attempted to interpret the best identified cluster structure using our new method, which is based on classifying each member in a cluster as either typical or atypical, as described in the introduction section of our paper.

All computations were performed using statistical software ROPstat (Vargha et al., 2015) and ROP-R (Vargha & Bánsági, 2022; Vargha & Grezsa, 2024).

## Results

### Basic statistics

Computing the basic statistics for the four scales, we found that all but one were significantly skewed at the *p*<.001 level, but the two avoidance scales only to a negligible extent (skewness < 0.50). However, the two anxiety scales were heavily skewed, with a skewness value above 1.5 and a median of 1, which is the lowest possible score on these scales (see Table 1).

**Table 1:** Basic descriptive statistics of the four attachment scales for complete cases (N = 752).

Scale	Median	Mean	SD	Minimum	Maximum	Skewness	Kurtosis
AvoidMo	3	3.37	1.76	1	7	0.40***	-0.88***
AnxMo	1	1.66	1.20	1	7	2.27***	5.11***
AvoidFa	4	4.01	1.78	1	7	0.10	-0.96***
AnxFa	1	1.85	1.39	1	7	1.89***	2.98***

*Notation*:

*** *p* < .001

### Confirmatory factor analysis (CFA)

The structural validity of the four parental attachment scales was examined by a CFA. In CFA we chose a robust method for model fitting (maximum likelihood mean variance, MLMV), which, in the case of CFA, provides a good alternative to the traditional ML method requiring multivariate normality (Gao et al., 2020). To assess the global fit of the CFA models, the following fit indices were used, with the cut-off values in parentheses: Comparative fit index – CFI (> .90), Tucker–Lewis index – TLI (> .90), root mean squared error of approximation – RMSEA (< .08), standardized root mean squared error – SRMR (< .08) (Hu & Bentler, 1999; van de Schoot et al., 2012).

A *χ^2^* test (*χ^2^* = 175.32, *df* = 48, *p* < .001) of the four-scale parental attachment factor model indicated an inadequate model fit. However, the significance of this test is highly sensitive to sample size, easily rendering the *χ^2^* test statistic significant (Alavi et al., 2020; Byrne, 2016). The other fit indices were all acceptable (RMSEA = .060, CI = [.51; .70], CFI = .950, TLI = .931, SRMR = .034), indicating a good fit.

### Reliability

The reliability level of each attachment scale is excellent (see Table 2).

**Table 2:** Alpha and omega reliability measures of the four attachment scales.

Scale	Usable cases	Alpha	CI_.95_	Omega	CI_.95_
AvoidMo	875	0.880	0.886-0.894	0.882	0.864-0.899
AnxMo	871	0.866	0.851-0.882	0.873	0.846-0.901
AvoidFa	777	0.865	0.849-0.882	0.878	0.860-0.896
AnxFa	758	0.888	0.875-0.902	0.893	0.871-0.916

Given the good fit of the four-factor CFA and the high reliability of the four parental attachment scales, they can be considered psychometrically suitable for exploring attachment types using CA. With a type here we mean a value profile that tends to occur frequently (Bergman et al., 2017). If a cluster is acceptably homogeneous, its members will have similar value patterns, and the cluster centroid will represent this common value pattern, called type. If the total sample is large enough, we can reasonably assume that a type identified in CA as a frequent value pattern also occurs in the population with a similar proportion.

### Standard hierarchical and *k*-means CA

Before performing any CA, for the sake of identifying multivariate outliers a residual analysis was performed proposed by Bergman (1988). This involves forming a residue from cases where the distance to the nearest neighbor exceeds a specified threshold prior to CA. For standardized variables the suggested threshold for being considered too extreme or outlier is 0.70 (Bergman, 1988; Bergman et al., 2003; Vargha et al., 2015). Since we did not want to standardize our variables before CA, to obtain a reasonable threshold we multiplied the suggested threshold of 0.70 by the mean variance of the variables submitted to CA. This yielded a threshold of 1.705. There was one single case for which the distance to the nearest neighbor (1.861) slightly exceeded this value. However, since this case's value pattern (maximum values for all variables) did not indicate a technical error, but rather an extremely negative attitude towards the person's parents, we decided not to exclude this case from the analysis.

Next an agglomerative hierarchical cluster analysis (AHCA) was run with Ward’s method. The range of the number of clusters was set between 2 and 10, and the standardization of the four attachment variables, having the same ranges of [1–7], was not asked before CA. A good help in the ROP-R output of AHCA is the summary table of some quality coefficients (QCs) for different AHCA cluster solutions (see Table 3). In this table, EESS% is a kind of explained variance percentage, the generalization of the eta effect size measure used in ANOVA, a measure of the homogeneity (coherence) of the cluster structure. XBmod is a measure of cluster separation and HCmeanS = HCmean/*Var_t_* is the standardized form of the HCmean cluster homogeneity coefficient (see Vargha et al., 2016; Bergman et al., 2017).

**Table 3:** Some QCs for different AHCA cluster solutions.

Number of clusters (*k*)	EESS%	XBmod	HCmeanS	HCstan min-max
10	76.0	0.37	0.49	0.19–1.44
9	74.2	0.28	0.52	0.19–1.44
8	72.4	0.22	0.56	0.28–1.44
7	69.6	0.14	0.61	0.28–1.80
6	66.7	0.56	0.67	0.28–1.80
5	62.8	0.51	0.75	0.28–1.80
4	53.5	0.45	0.93	0.28–1.80
3	43.0	0.33	1.14	0.28–2.05
2	28.4	0.50	1.43	0.28–1.86

*Note*. EESS% = Explained error sum of square percentage; XBmod = Modified Xie-Beni index; HCmeanS = average *z_t_*-standardized within cluster difference.

Table 3 shows that if the cluster number (*k*) is less than 6, EESS% is below 65%, the lower acceptable level of overall cluster homogeneity (Bergman et al., 2003; Vargha et al., 2016); *k* = 6 is also the cluster number where the XBmod measure of cluster separation (Bergman et al., 2017) reaches its maximum. Therefore, an optimal number of clusters in our sample may be somewhere around *k* = 6.

Next, based on the AHCA results, standard *k*-means cluster analyses were performed with the number of clusters set to between 5 and 7. As before, the variables were not standardized prior to CA, just as in AHCA. The computed QC values of these solutions, summarized in Table 4, show that the *k* = 6 solution is the most attractive showing a good level of both overall homogeneity (EESS% ≈ 70) and cluster separability (XBmod > 0.5, SC > 0.5; see Vargha et al., 2016). In addition, using the *Validation* module in ROPstat, a data simulation was undertaken to verify that the QC values in Table 4 were higher than what could be expected on a random data set with similar general properties (same number of clusters, same number of variables, same intercorrelations between variables) as the data set used in the real analysis. All QCs for all three number of clusters were significantly higher than the averages of 25 independent replications of simulated values. The relative improvements of the QCs of the real data against the simulated ones can be measured by the MORI coefficients (Vargha et al., 2016) and these are summarized for the three number of clusters in Table 5. As can be seen in this table, MORI values for the QCs that measure overall homogeneity (EESS% and HCmeanS) are best for *k* = 6 and *k* = 7 at around MORI = 25%. Meanwhile, the MORI values for the QCs that measure also cluster separability (PB, XBmod and SC, see Bergman et al. 2017) are best uniquely for *k* = 6, with values ranging from 17% to 33%. This result also confirms that the 6-cluster solution should be accepted as the best cluster structure.

**Table 4:** Some QCs of k-means solutions for cluster numbers between k = 5 and k = 7.

Number of clusters	EESS%	PB	XBmod	SC	HCmeanS	HCstan min-max
5	66.1	0.44	0.51	0.66	0.68	0.33–1.70
6	69.9	0.44	0.55	0.66	0.61	0.34–1.71
7	72.4	0.38	0.25	0.61	0.56	0.20–1.63

*Note*. EESS% = Explained error sum of square percentage; PB = cluster point biserial correlation; XBmod = Modified Xie-Beni index of cluster separability; SC = Simplified Silhouette coefficient; HCmeanS = average *z_t_*-standardized within cluster difference.

**Table 5:** MORI coefficients for some QCs of k-means solutions for cluster numbers between k = 5 and k = 7.

Number of clusters	EESS%	PB	XBmod	SC	HCmeanS
5	0.22	0.14	0.19	0.29	0.22
6	0.25	0.17	0.33	0.31	0.25
7	0.26	0.10	-0.23	0.21	0.26

*Note*. EESS% = Explained error sum of square percentage; PB = cluster point biserial correlation; XBmod = Modified Xie-Beni index of cluster separability; SC = Simplified Silhouette coefficient; HCmeanS = average *z_t_*-standardized within cluster difference.

**Figure 1:**
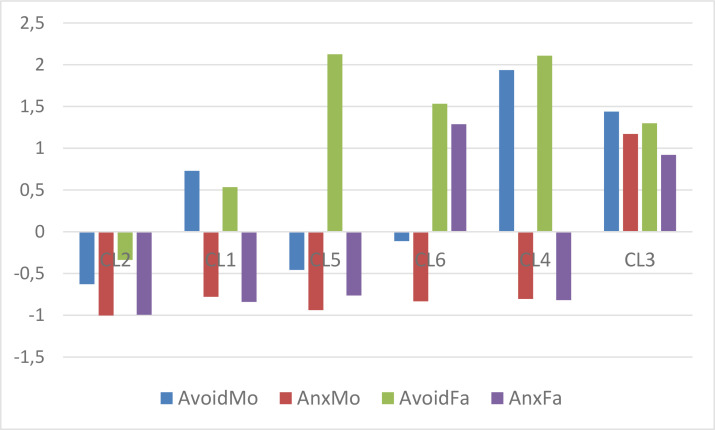
The z_t_-standardized centroid pattern of the 6-cluster k-means solution (clusters arranged in descending order of overall attachment level)

**Figure 2:**
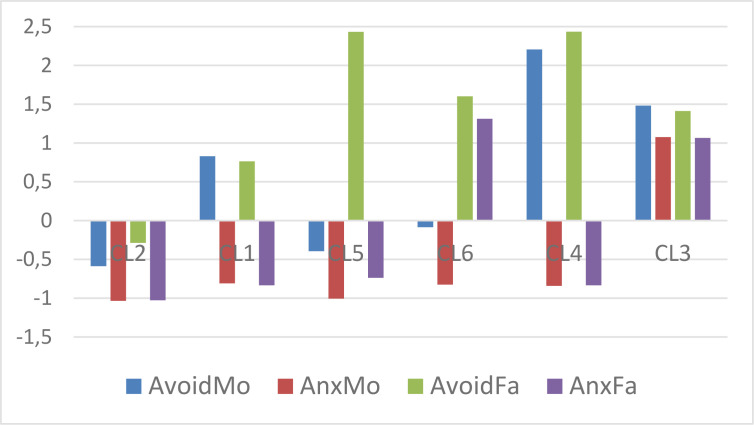
The z_t_-standardized centroid pattern of the 6-cluster k-means solution without atypical cases (clusters arranged in descending order of overall attachment level)

The pattern of centroids of the 6-cluster *k*-means solution based on *z_t_*-standardized means can be assessed by inspecting Table 6 and Figure 1. The *z_t_*-standardization is a method for illustrating the centroids of a CA solution, when we do not standardize before CA (Vargha, 2025). This can only be applied if the variables have the same scale. Otherwise, the usual *z*-standardization or some other type of normalization (e.g., POMS transformation, see Moeller, 2025) is unavoidable. Having identical scales, how can we decide, after performing a CA, which cluster mean should be considered low or high? The answer is to subtract from each cluster mean for each variable a common mean level (the *M_t_* average of the variables in the total sample) and divide this difference by a common SD (the square root of the mean of the variances), denoted by *SD_t_*. This is called *z_t_*-standardization, and it provides a tool for interpreting the cluster centroids in a common framework, where high and low variable levels can easily be grasped (see, e.g., Figure 2).

**Table 6:** The pattern of *z_t_*-standardized means in the 6-cluster *k*-means solution (H = High, L = Low; more pluses indicate more extreme means).

Cluster	AvoidMo	AnxMo	AvoidFa	AnxFa	CLsize	HCstan
CL1	H	L	(H)	L	197	0.52
CL2	(L)	L+	.	L	231	0.34
CL3	H++	H+	H+	H	72	1.71
CL4	H+++	L	H++++	L	129	0.53
CL5	(L)	L	H++++	L	72	0.47
CL6	.	L	H++	H+	51	0.93

Table 6 and Figure 1 indicate two trivial types (CL2, representing the type of best parental attachment and CL3, representing the worst), where all attachment means depart from the overall mean in the same direction, and four nontrivial types, where special patterns of means emerge (e.g., CL1, representing a type where both avoidance scales are high and both anxiety scales are low, or CL5, representing a type where all scales are low except Father avoidance, which is very high).

### Typical and atypical cases

One nice feature of the obtained cluster structure is that each cluster has a relevant psychological meaning. However, there is one issue: cluster CL3 is very heterogeneous (HCstan = 1.71; see Table 6). Does the centroid of CL3, as shown in Table 6 and Figure 1, accurately represent its type? Would disregarding atypical cases of this cluster change the pattern and meaning of CL3?

The latest version of ROPstat’s *Validation* module in the *Pattern-oriented analysis* menu can compute and save the average squared Euclidean distance (ASED) from its own cluster centroid for each case for a specified cluster code variable. If standardization is requested, this distance is computed based on *z*-standardized scores. If standardization is not requested, the distance is computed based on *z_t_*-standardized scores. This new variable, which is added to the data table on request, is named after the cluster code variable and has the letter 'd' added to the end. In addition, the typical and atypical groups will be defined for this variable by means of the threshold value of 0.70.

The pattern of *z_t_*-standardized means in the 6-cluster *k*means solution computed in the subsample of typical cases (*N* = 687) can be seen in Table 7. The *z_t_*-standardization was performed by means of the common mean and the mean variance of variables restricted to typical cases. In Table 7 a cell is highlighted in light grey if it differs from the corresponding cell identified for all cases in Table 6. Although a change can be seen in seven cells when Tables 7 and 6 are compared, all these changes are slight. This suggests that the overall pattern of centroids did not change substantially, which is also reflected by Figure 2.

**Table 7:** The pattern of z_t_-standardized means in the 6-cluster k-means solution in the subsample of typical cases (H = High, L = Low; more pluses indicate more extreme means; cells that differ from the corresponding cell identified for the total sample in Table 6 are highlighted in light grey).

Cluster	AvoidMo	AnxMo	AvoidFa	AnxFa	CLsize	HCstan
CL1	H	L	H	L	184	0.49
CL2	(L)	L+	.	L+	227	0.38
CL3	H++	H+	H++	H+	41	0.83
CL4	H++++	L	H++++	L	123	0.57
CL5	.	L+	H++++	L	70	0.50
CL6	.	L	H++	H+	42	0.85

The types of parental attachment explored (see Figure 2) are psychologically meaningful. CL2 represents the best type of parental attachment (with low levels of parental avoidance and anxiety), while CL3 represents the worst (with high levels of parental avoidance and anxiety). CL1 and CL4 both represent types where the parental attachment is poor in terms of avoidance (high levels), but good in terms of anxiety (low levels). However, CL4 shows a higher level of parental avoidance. CL5 represents a fundamentally positive attachment, like CL2, where only the high level of Father avoidance indicates an issue with the paternal attachment. Finally, CL6 represents a type of attachment where the maternal attachment is good (average avoidance coupled with low anxiety), whereas paternal attachment is poor (high avoidance coupled with high anxiety).

The main features – numbers, percentages and HCstan values – of typical and atypical cases in the different clusters are summarized in Table 8. To ensure a valid comparison, the HCstan values for both typical and atypical cases were computed based on the mean variance of the variables in the total sample (*N* = 752), rather than the restricted samples (as with the typical cases in Table 7).

**Table 8:** The number and percentage of typical and atypical cases in the different clusters, as well as HCstan values

Cluster	Typical cases			Atypical cases			Together

	Number	Percentage	HCstan	Number	Percentage	HCstan	HCstan
CL1	184	93.4	0.40	13	6.6	1.11	0.52
CL2	227	98.3	0.31	4	1.7	0.59	0.34
CL3	41	56.9	0.68	31	43.1	2.95	1.71
CL4	123	95.3	0.47	6	4.7	1.32	0.53
CL5	70	97.2	0.41	2	2.8	0.20	0.47
CL6	42	82.4	0.70	9	17.6	1.24	0.93

Based on the data of Table 8 we can test whether the percentage of atypical cases have approximately the same rank order as the size of the heterogeneity coefficients. Spearman's rho was computed for these two variables across the six clusters, yielding a value of *rho* = .943, which indicates a very strong relationship.

If the homogeneity of a cluster of typical cases is not good enough (say HCstan is not less than 0.50), we are reluctant to consider the centroid as a type. In our data, CL3 and CL6 fall into this category (see Table 8). However, considering some specific circumstances, we can be a little permissive. In the case of CL3 we can say that it contains persons with high maternal and paternal anxiety values (see Figure 2), which is fairly rare in the total sample (more than 50% of the persons in the total sample can be characterized with the minimum value of these two variables; see the medians in Table 1). This means that there will be high variability in parental anxiety in CL3, yielding that members in CL3 will differ slightly from each other, but all can be labelled with high parental anxiety – and also a similarly high level of parental avoidance, which also occurs in some other clusters (e.g., CL1 and CL4, see Figure 2). Therefore, we feel justified accepting the centroid of CL3 restricted to typical persons as a type, and with a similar reasoning the centroid of CL6. To conclude, the centroid of each restricted cluster in our sample can be considered a type.

Though Table 7 and Figure 2 suggest that atypical cases have negligible influence on the overall cluster pattern, it is worth seeing how the means of atypical cases differ from the means of typical cases.

**Figure 3:**
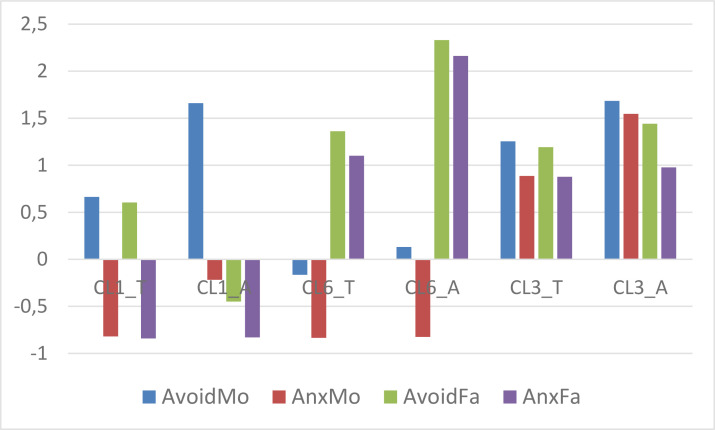
The z_t_-standardized centroid pattern of CL1, CL3 and CL6 plotted separately for the subclusters of typical (T) and atypical (A) cases

The proportion of atypical cases exceeded 5% in three clusters: in CL1, CL3, and CL6 (see Table 8). For these clusters we computed and plotted centroids separately for the typical and atypical cases (see Figure 3) and tested for each variable the difference between means via Welch’s *t*-test (Welch, 1947), which does not assume variance homogeneity (see Table 9). For other clusters it makes no sense to go into further detail since the number of atypical cases is very low.

**Table 9:** Comparison of cluster means of typical (T) and atypical (A) cases within clusters CL1, CL3 and CL6 for the four input variables via Welch’s t-test

Variable	Welch *t*	*df*	*p*-value	Cohen-*d* effect size^a^
*CL1_T versus CL1_A*				
AvoidMo	3.88	12.5	.002**	1.82
AnxMo	2.46	12.4	.029*	1.27
AvoidFa	6.90	13.3	<.001***	–2.27
AnxFa	0.07	12.8	0.946	0.03
*CL3_T versus CL3_A*				
AvoidMo	1.96	39.2	.057+	0.51
AnxMo	3.41	47.8	.001**	0.86
AvoidFa	0.99	37.9	0.329	0.26
AnxFa	0.37	40.0	0.715	0.10
*CL6_T versus CL6_A*				
AvoidMo	0.73	9.3	0.485	0.36
AnxMo	0.06	9.7	0.956	0.03
AvoidFa	4.58	12.2	.001***	1.62
AnxFa	4.11	10.7	.002**	1.66

*Notation*:

+ : *p* < .10

* : *p* < .05

** : *p* < .01

*** *p* < .001

a : Cohen’s *d* is positive if the mean of the atypical group is higher, and negative if it is lower.

Based on Figure 3 and Table 9 we can draw the following conclusions.

- In CL1 three out of four variables differ between typical and atypical cases, only the level of Father anxiety is the same; in the atypical subgroup Mother avoidance and Mother anxiety are substantially higher, while Father avoidance is substantially lower, with Cohen-*d* values all above 1.
- In CL3 only the levels of Mother anxiety differ (in the atypical subgroup it is substantially higher with a Cohen-*d* value of 0.86).
- Finally, in CL6 only the levels of paternal attachment scales differ (in the atypical subgroup Father avoidance and anxiety levels are substantially higher, with Cohen-*d* values above 1.5).

### Gender and age impacts

As an extension of our interpretation of the 6-cluster solution, we investigated the relationship of this clustering with gender and age. In the case of gender, the relationship was strong (Cramér’s *V* = 0.21, χ*^2^* = 33.71, *df* = 5, *p* < .001) due to one single cluster, CL4, in which men (60.5%) were dominant over women, compared to their proportion in the total sample (42.3%). Regarding age, no statistically significant or interpretable result was obtained. We also compared typical and atypical cases in terms of age and gender but did not obtain any significant results at the *p* < .05 level. This may also be due to the small sample sizes of the atypical groups, of course.

## Discussion

Heavily skewed variables can cause problems in CAs. If a variable is heavily skewed, it will have a long tail, with increased distances between cases in this part of the distribution. The increased distances may yield heterogeneous clusters, deteriorating the overall homogeneity of the cluster structure, but of course, cluster heterogeneity can also be the consequence of other factors.

The aim of the present paper was to propose a method for interpreting cluster analysis results in the presence of heterogeneous clusters. The key concept is a new variable that measures how far each case is from its own cluster center, after the CA has been conducted. A case is considered typical if this distance does not exceed a predetermined threshold, and atypical if it does. As a rule, this threshold is 0.70 for *z*-standardized variables, or, equivalently, 0.70 times the mean of the variances of the input variables if they have the same range of values.

The usefulness of this method was demonstrated using four parental attachment variables, where high skewness and heterogeneous clusters are anticipated. The study sample consisted of 918 young adults aged between 20 and 35, where the percentage of women was 57.5%.

In the statistical analyses we checked first the factor structure of the parental attachment scales of a newly shortened ECR-RS via CFA, where the model fit was good. The computed Cronbach’s alpha and McDonald’s omega reliability measures indicated high level reliability. Then we conducted standard hierarchical and *k*-means CAs to explore a statistically adequate and theoretically acceptable cluster structure of parental attachment. Here we identified a 6-cluster structure as the best solution.

The types of parental attachment explored (see Table 6 and Figure 1) were psychologically meaningful. One cluster (CL2) represented the best type of parental attachment with low levels of avoidance and anxiety, and another one (CL3) represented the worst type. Two clusters (CL1 and CL4) represented types where the parental attachment was poor in terms of avoidance, but good in terms of anxiety (CL4 showing a higher level of parental avoidance). One cluster (CL5) represented a fundamentally positive attachment, where only the high level of father avoidance indicated an issue with parental attachment. Finally, one cluster (CL6) represented a type of attachment where the maternal attachment was good (average avoidance coupled with low anxiety) and the paternal attachment poor (high avoidance coupled with high anxiety).

Finally, we classified each case as either typical or atypical depending on how far the case was from its own cluster centroid (using the *Validation* module of the latest version of ROPstat). The overall pattern of centroids did not change substantially when they were computed for typical cases only, but several minor changes could be detected. Comparing typical and atypical cases in the clusters where the proportion of atypical cases exceeded 5%, significant and well explainable differences were obtained (see Table 9). Summarizing, the idea of classifying each case as either typical or atypical, depending on how far it is from its own cluster centroid, has proven useful for obtaining a more refined interpretation of the cluster structure.

An unexpected, interesting finding is that the subgroup of atypical cases can have some relevance even in relatively homogeneous clusters, such as CL1, where HCstan was 0.52. This small but not negligible group (*n* = 13; 6.6% of the entire cluster) differed from the group of typical cases with respect to three out of four input variables. This means that our proposed new method is worth applying not only to cluster structures where heterogeneous clusters occur, but also to structures where one or more clusters are moderately homogeneous.

To better explain the meaning of the six-cluster solution, we investigated the relationship between this clustering and gender and age. Only gender had an impact, due to a cluster (CL4), where low parental anxiety was coupled with very high parental avoidance. The proportion of men in this cluster was significantly higher (60.5%) than in the total sample (42.3%).

The parental attachment styles explored were comparable to those found in the literature. Building on the same two-dimensional framework of anxiety and avoidance, and the separate consideration of mothers and fathers as in the cited studies, our six-cluster, person-centered solution recovers recognizable, theoretically coherent types. The cluster marked by globally low avoidance and low anxiety across both parents maps onto the secure pattern: in prior work, secure individuals describe parents in more benevolent, less punitive, and better differentiated ways, with a higher conceptual level in representations of both mother and father (Levy et al., 1998). On the process side, lower maternal avoidance is indirectly linked to greater secure base use in adolescence, through lower parental hostility and more positive adolescent perceptions (Jones & Cassidy, 2014).

At the opposite pole, the cluster of worst attachment with concurrently high avoidance and high anxiety resembles a fearful or preoccupied composite. This configuration has been associated with more punitive or ambivalent parental representations, while the fearful pattern can remain relatively well differentiated despite its negative tone (Levy et al., 1998), and with less favorable secure base processes (Jones & Cassidy, 2014).

Two additional clusters trace a dismissing-like continuum defined by elevated avoidance with low anxiety in relation to both parents. The moderate-avoidance variant corresponds to the classic dismissing profile, characterized by lower conceptual level, poorer differentiation, and fewer benevolent attributes in mother and father representations (Levy et al., 1998), and it dovetails with the indirect, maternal-avoidance-driven reduction in adolescents’ secure base use (Jones & Cassidy, 2014). The more extreme-avoidant variant is interpretable as an “extreme dismissing” type, that is, the same content pattern in amplified form. Both variants are consistent with prior descriptions of avoidant-dominant configurations.

Finally, the types of two asymmetric clusters reproduce dissociations between mothers and fathers reported else-where and are consistent with the process specificity found for fathers. In one of them (“good mother–avoidant father” with high paternal avoidance and low paternal anxiety), risk is expected to be comparatively lower, because paternal anxiety, rather than avoidance, emerges as the primary driver of the sequence from parental hostility to adolescent perceptions to reduced secure base use. This interpretation is also in line with the review’s conclusion that avoidance has emerged as the dominant predictor of less observed parental sensitivity, while observational work with fathers remains scarce, warranting cautious father-specific inferences. In contrast, in the “good mother–problematic father” cluster (with high paternal avoidance and high paternal anxiety) one should entail higher risk, as high paternal anxiety is likely to potentiate that indirect route (Jones & Cassidy, 2014). Typologically, multi-type solutions extracted from the same four parent-specific ECR-RS scales in large adolescent samples also isolate asymmetric mother–father combinations and avoidant-dominant types (Vargha & Grezsa, 2024; Vargha, 2025), providing additional content-level corroboration that our six-type partition identifies valid, literature-convergent attachment profiles.

## Data Availability

The research data analyzed in this paper are available from the corresponding author on request.

## Action editor

Lars-Gunnar Lundh served as action editor for this article.

## Open access

This article is distributed under the terms of the Creative Commons Attribution 4.0 International License (http://creativecommons.org/licenses/by/4.0/), which permits unrestricted use, distribution, and reproduction in any medium, provided you give appropriate credit to the original author(s) and the source, provide a link to the Creative Commons license, and indicate if changes were made.
